# Global change-driven plant adaptive strategies and interspecific interaction networks: from molecular regulation to ecological management

**DOI:** 10.3389/fpls.2026.1904136

**Published:** 2026-09-15

**Authors:** Qing Liu, Ya Zhang, Ruixi Zhang, Junxia Zhou

**Affiliations:** School of Life Sciences, Henan University, Kaifeng, Henan, China

**Keywords:** biological invasion, ecosystem management, global change, interspecific interactions, multiple stresses, plant adaptation

## Abstract

Global change drivers, including climate warming, elevated CO_2_, nitrogen deposition, extreme climate events, heavy metal pollution, and biological invasions, interact in complex ways to reconfigure terrestrial ecosystems. This review synthesizes recent advances to develop a framework linking plant molecular responses to ecosystem management. We first examine how abiotic stressors reshape plant physiology and traits through defense signaling, secondary metabolite synthesis, and life-history adjustments. We then analyze how these plant-level changes cascade upward to restructure interspecific networks, focusing on plant-insect and plant-microbe dynamics mediated by altered chemical communication and resource competition. The mediating role of soil biogeochemistry and plant-soil feedback is also considered. Building on this mechanistic understanding, we propose that global change does not uniformly facilitate plant invasion; rather, it differentially filters invasive lineages along three axes: resource availability (elevated CO_2_, nitrogen deposition), disturbance regime (extreme drought-rewetting, warming amplitude), and life-history strategy (annual vs. perennial, sexual vs. clonal). Seed functional traits and climate-driven shifts in reproductive phenology operate as the upstream drivers that preconfigure interspecific network structure. Finally, we outline a management framework organized around four invader mechanism types: resource-acquisitive, plasticity-dependent under warming, drought-rewetting adapted, and microbiome-dependent, each matched with empirically validated, scenario-specific interventions. Integrating functional trait data with species distribution models enables quantitative forecasting of distribution shifts and identification of ecologically vulnerable zones. Such predictive outputs provide the data basis for full-cycle ecological management, from risk warning to targeted habitat regulation, offering a scientific foundation for biodiversity conservation and ecosystem restoration under global change.

## 1 Introduction

Currently, Earth’s ecosystems are undergoing profound transformations driven by human activities. The increase in atmospheric CO_2_ concentration, global warming, frequent extreme climate events, intensified nitrogen deposition, the spread of heavy metal pollution, and the large-scale cross-regional migration of biological species together constitute a complex and interconnected stress matrix of “global change” (Zhang et al., 2023c, 2022). As the primary producers and structural foundation of ecosystems, plants are profoundly affected in their survival, growth, reproduction, and interactions with other organisms. Understanding how plants perceive, integrate, and respond to multiple stress signals, thereby adjusting their physiological, morphological, life-history, and chemical defense strategies, has become a core scientific question for predicting the future trajectory of ecosystems (Muhammad et al., 2024; Kang et al., 2019).

Crucially, these adaptive adjustments of plants cannot occur independently of external environments. They continuously transmit changing “chemical language” to surrounding herbivores, pollinators, competitors, and soil microorganisms by releasing volatile organic compounds, altering root exudates, and adjusting tissue nutrient composition (Liu et al., 2022). The reprogramming of this chemical signaling directly drives the reconstruction of interspecific interaction networks. For instance, climate warming may alter insect oviposition preferences toward invasive versus native species (Liu et al., 2022), whereas heavy metal pollution can grant some invasive species elemental defense superiority and trigger apparent competition against native plants (Wang et al., 2022b). Simultaneously, invasive plants themselves exhibit remarkable adaptive plasticity, with seed traits and defensive strategies undergoing rapid divergence across environmental gradients to acclimate to new habitats (Zhou et al., 2021). These dynamics, occurring at the individual, population, and community levels, ultimately affect ecosystem material cycling and energy flow through pathways such as plant-soil feedback (Feng et al., 2023; Yu et al., 2022). In invasion biology, “biological invasion” describes alien plants, animals and pathogens establishing self-sustaining populations and damaging recipient ecosystems (Richardson and Pyšek, 2008). For clarity, this review exclusively focuses on invasive plants; herbivorous insects, natural enemies and pathogenic microbes are only covered as biotic stressors that interact with plants to regulate their defensive signaling and rhizosphere feedback, rather than independent invasive taxa.

Therefore, investigating stress physiology, chemical ecology, or invasion biology in isolation is no longer adequate to tackle contemporary ecological challenges. This review systematically synthesizes published research to uncover how global change drivers alter connectively patterns across the entire ecological network. This understanding is not only a deepening of theoretical ecology but also the cornerstone for developing intelligent, proactive, and predictive ecological management strategies.

## 2 Reshaping of plant physiology and traits by abiotic stress

Abiotic factors in the context of global change, particularly climate warming and environmental pollution, affect individual plants at unprecedented intensity and scope. These factors trigger cascaded adaptive responses spanning molecular sensing, physiological metabolism and phenotypic traits. This adaptive adjustment represents no passive damage response, but the activation and restructuring of inherent survival strategies conserved throughout plant evolutionary history, which can be initiated upon perception of environmental signals. Its core lies in the plant’s ability to integrate specific stress signals and achieve re-matching with the changing environment at the individual and population levels by adjusting resource allocation, reconstructing metabolic networks, and modifying chemical phenotypes (**Figure 1**). In-depth analysis of the underlying mechanisms and external ecological responses of this adaptive regulation is key to predicting the future dynamics of plants and even entire ecosystems.

**Figure 1:**
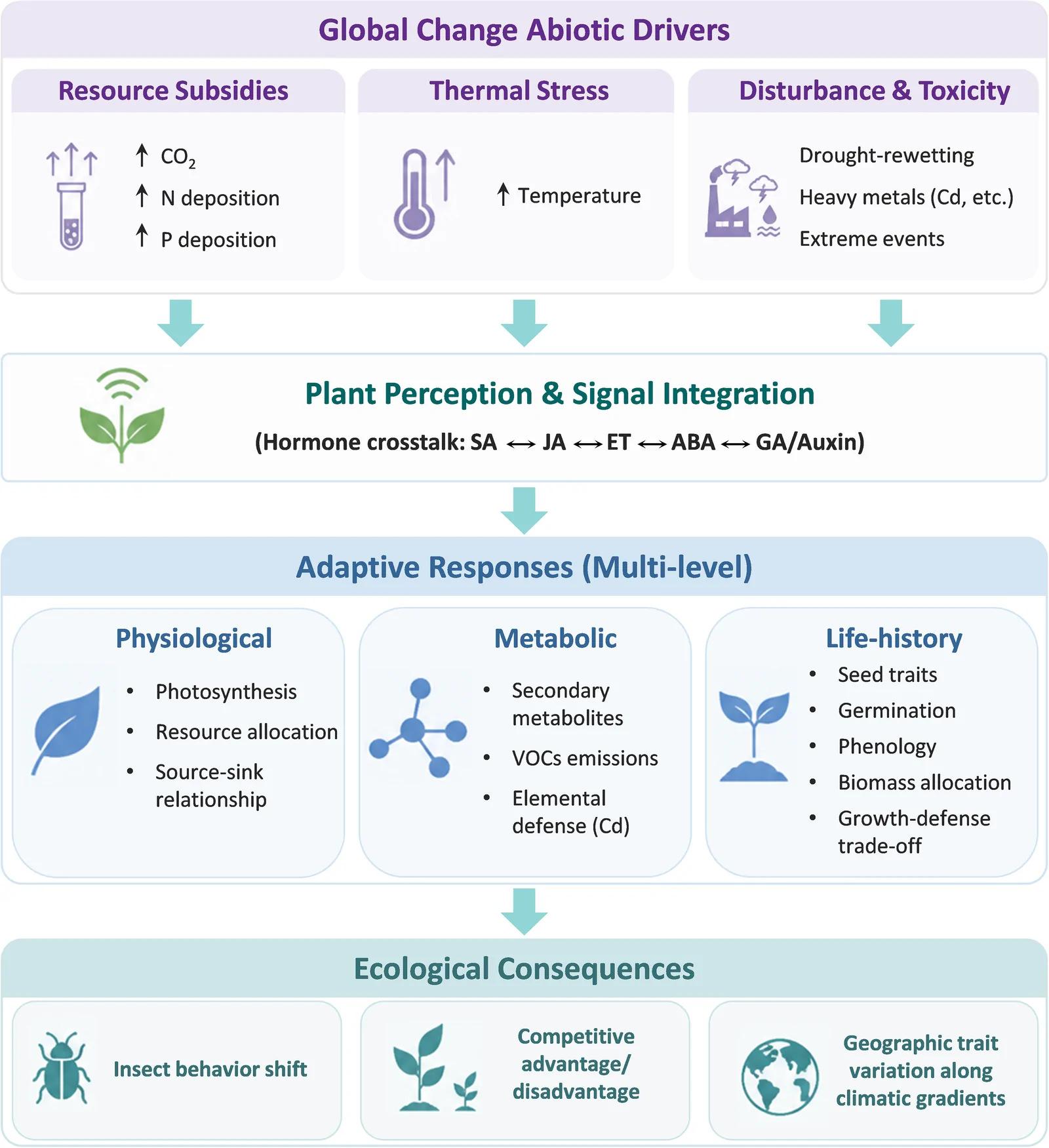
Conceptual framework for the reshaping of plant physiology and traits under global-change abiotic stress. Three classes of abiotic global-change drivers (resource subsides, thermal stress, and disturbance/toxicity) activate plant signal integration via hormone crosstalk. This cascade drives physiological, metabolic and life-history adaptive responses, which further produce ecological consequences such as shifts in insect behavior, altered plant competitive performance, and climate-associated geographic trait variation. Symbol explanations: “↑” indicated elevation; “↔” indicated interaction. The N represented nitrogen; P represented phosphorus; Cd represented cadmium; SA represented salicylic acid; JA represented jasmonic acid; ET represented ethylene; ABA represented abscisic acid; GA represented gibberellin; VOCs represented volatile organic compounds.

### 2.1 Warming-mediated modulation of plant chemical phenotype and insect behavior

As one of the most significant abiotic stress factors, global climate warming is profoundly and delicately reshaping plant physiological metabolism and phenotypes, thereby triggering cascading changes in the behavioral responses of herbivorous insects and ultimately reshaping plant-insect interaction networks (Liu et al., 2022; Luo et al., 2020; Wu and Ding, 2019; Lu et al., 2019). Rather than following a simple linear pattern, this reprogramming process constitutes intricate biological crosstalk. The entire cascade includes temperature signal perception, modulation of secondary metabolism, emission of specific volatile organic compounds (VOCs) and shifts in tissue chemistry, all of which shape insect olfactory behavior, host selection, oviposition and development (Martin-Cacheda et al., 2025; Xiao et al., 2024; Yu et al., 2022; Wang et al., 2020a; Xiao et al., 2019).

Studies have demonstrated that experimental warming can significantly alter the composition and relative proportions of leaf VOCs in the invasive plant *Alternanthera philoxeroides* and its native congener *A. sessilis* (Liu et al., 2022). This reprogramming of the chemical fingerprint indicates that the chemical signals released by plants into the environment undergo fundamental changes under elevated temperatures (Yu et al., 2022; Wang et al., 2020a; Xiao et al., 2020). For herbivorous insects that rely on these chemical signals for host location, such changes may render the original olfactory cues ineffective or distorted (Martin-Cacheda et al., 2025; Liang et al., 2025; Wang et al., 2020a). For example, behavioral experiments on the generalist pest *Spodoptera litura* revealed an asymmetric effect: under warming conditions, its oviposition preference for native *A. sessilis* showed a relative increase, while its olfactory orientation toward invasive *A. philoxeroides* did not change significantly (Liu et al., 2022). The ecological consequences of this modulation are profound and subtle. Under global warming, the damage pressure from generalist pests may shift systematically from certain plant groups (e.g., some invasive plants) to other groups (e.g., native plants) (Zhou et al., 2022; Luo et al., 2020; Lu et al., 2019). This shift is not random but is directionally guided by warming-induced variations in plant chemical phenotypes (Xiao et al., 2024; Wang et al., 2020a). The potential result is that some native plants may suffer decreased fitness due to additional herbivore pressure, while some invasive plants may gain a competitive advantage by being relatively “relieved” of part of the herbivore pressure, potentially accelerating their invasion process (Liu et al., 2022; Luo et al., 2020; Wu and Ding, 2019; Lu et al., 2019). The sequential functional cascade from warming to plant chemical phenotypic shifts and subsequent shifts in insect oviposition preference clearly demonstrates that climate change modulates hidden interspecific chemical signaling pathways, thereby asymmetrically altering species interaction balances in ecological communities (Martin-Cacheda et al., 2025; Wang et al., 2020a). It transcends the traditional view of warming as merely a growth-limiting factor, elevating it to an important driver of interspecific relationships within ecosystems (Liu et al., 2022; Tian et al., 2022; Zhang et al., 2021a). Understanding this regulatory logic is of key significance for predicting trends in agricultural pest outbreaks, the effectiveness of biological control agents, and the succession direction of plant communities in natural ecosystems under future climate scenarios (Liu et al., 2022; Wang et al., 2020a; Lu et al., 2019). Additionally, warming may also indirectly regulate the intensity and composition of constitutive and induced defenses by affecting plant primary metabolism and resource allocation, thereby altering the precursor supply for synthesizing defensive compounds (Xiao et al., 2024; Liu et al., 2022; Xiao et al., 2019).

This multi-level, multi-channel regulation effect makes the impact of warming on plant-insect interactions highly complex and context dependent (Liu et al., 2022; Luo et al., 2020; Lu et al., 2019). Therefore, future research needs to integrate multidisciplinary approaches from plant physiological ecology, chemical ecology, insect behavior, and community ecology to analyze the molecular mechanisms and ecological network cascade effects of warming-induced plant-insect interactions under more realistic compound stress scenarios (e.g., warming combined with drought, elevated CO_2_, extreme climate events) (Liu et al., 2022; Wu and Ding, 2019; Henriksen et al., 2018; Pan et al., 2026; Zhang et al., 2025a). This is not only a scientific necessity for addressing biodiversity conservation and agricultural sustainability under global change but also a core component for understanding the feedback mechanisms of ecosystem responses to climate change (Miao et al., 2020; Luo et al., 2020; Wu and Ding, 2019).

### 2.2 Resource subsidies from elevated CO_2_ and nitrogen deposition

Among the abiotic drivers of global change, rising atmospheric CO_2_ concentration and accelerating nitrogen deposition act primarily as resource subsidies rather than as direct stressors. Their invasion-level consequences emerge not from toxicity or tissue damage, but from altered resource availability that magnifies the growth–defense trade-off in favor of invasive lineages. A phylogenetically controlled meta-analysis established that elevated temperature and CO_2_ enrichment enhance the performance of invasive alien plants more strongly than that of native plants, while invasive species also show a tendency (though statistically weaker) toward a more positive response to increased N deposition (Stephens et al., 2019). This asymmetry positions CO_2_ and N deposition as selective filters that disproportionately benefit certain invasion mechanisms.

The mechanistic basis of this filter has been quantified in open-top chamber experiments. Under elevated CO_2_, nitrogen addition alone raised the relative growth rate (RGR) of invasive *Mikania micrantha* and *Chromolaena odorata* by 5.4%; the combined effects of N addition plus elevated CO_2_, or P addition plus elevated CO_2_, further increased RGR by 7.5% and 8.1% respectively, reaching a 14.4% advantage over co-occurring native congeners (Zhang et al., 2024a). Concurrently, leaf chemical allocation shifted decisively toward growth: proteins rose by 75.7%, total non-structural carbohydrates by 8.5%, and minerals by 9.6%, while defense-related compounds including lipids and total structural carbohydrates, declined by 17.7% and 29.0%, respectively (Zhang et al., 2024a). These data demonstrate that resource subsidies do not merely accelerate invader growth; they re-allocate the invader’s metabolic budget away from defense and toward biomass accumulation, sharpening the competitive edge over slower-responding natives.

The competitive consequences manifest rapidly in multi-species mixtures. Using *Ambrosia artemisiifolia* and *Amaranthus retroflexus*, two notorious invasive annuals, as study materials grown in monospecific and mixed stands under factorial combinations of elevated CO_2_ and increased N, elevated CO_2_ combined with N addition increased shoot, root and total biomass of *A. artemisiifolia* and reinforced its competitive superiority, whereas elevated CO_2_ inhibited the root biomass of *A. retroflexus* (Zhao et al., 2022a). In other words, resource subsidies do not uniformly facilitate all invasive taxa; they differentiate among invaders according to life-history and resource-use strategy (Luo et al., 2020). Similarly, simulated N deposition increased the biomass, structural defense, and herbivore resistance of the invasive Alternanthera philoxeroides relative to its native congener *A. sessilis*, and magnified the invader’s competitive advantage irrespective of herbivore pressure (Lin et al., 2024). Together these results indicate that the invasiveness of resource-acquisitive species is amplified under enriched resource regimes, while stress-tolerant or slowly responding natives lose ground.

Below-ground, N deposition interacts with warming to restructure the rhizosphere microbial consortium that underpins nutrient acquisition. In the invasive *Ambrosia trifida*, warming and N addition together promoted biomass accumulation and enhanced soil nutrient content; warming increased the abundance of functional genes involved in carbon fixation, phosphorus mineralization and inorganic phosphate solubilization, and enriched beneficial taxa such as *Sphingomicrobium*, *Massilia* and *Nocardioides*, whereas N deposition exerted a comparatively weaker effect on the functional microbial community (Xu et al., 2026). This hierarchy (warming > N deposition) in shaping rhizosphere functioning implies that under future climates, resource subsidies will propagate through plant–microbe networks to compound invader competitive advantage (Yu et al., 2022).

At the biogeochemical scale, the proliferation of invasive plants fed by CO_2_ and N subsidies feeds back to the atmosphere. A global meta-analysis of 104 studies reported that plant invasion significantly increased soil emissions of CO_2_, CH_4_ and N_2_O, with C_4_ invaders driving larger enhancements than C_3_ invaders (Gu et al., 2025). Thus, resource subsidies and invasion success form a positive feedback loop: elevated CO_2_ and N deposition fuel invader growth, and the resulting invasion alters soil microbial processes to release additional greenhouse gases, which in turn accelerate global change.

### 2.3 Extreme climate events and plant adaptation during invasion

Beyond gradual warming, extreme climate events such as severe drought, abrupt rewetting, and heat waves are becoming more frequent under global change and exert distinct, often non-linear, effects on plant invasion. Unlike chronic resource subsidies (elevated CO_2_, N deposition), extreme events act as pulse disturbances that select for invaders with high phenotypic plasticity and rapid recovery capacity. Understanding how invasive plants exploit these pulses is central to predicting invasion dynamics under future climate scenarios.

Drought–rewetting pulses. A controlled greenhouse experiment with 14 plant species (6 invasive, 8 native) from three families (*Asteraceae*, *Amaranthaceae*, *Poaceae*) demonstrated that invasive species produced significantly more above- and below-ground biomass than natives under drought–rewetting cycles (Pan et al., 2026). Annual invaders outperformed perennials in above-ground biomass, whereas perennials allocated more biomass to roots, especially under drought (Pan et al., 2026). Rewetting reversed drought suppression and favored invaders, with above-ground biomass increasing more in invasives than in natives (Pan et al., 2026). These results establish a clear pattern: drought–rewetting pulses act as a disturbance filter that selectively benefits invasive lineages, particularly those with annual life histories and rapid resource-acquisition strategies.

Extreme temperature and reproductive phenology. Experimental warming (+1.5 °C across two growing seasons) applied to the invasive C_4_ grass *Spartina alterniflora* in a coastal salt marsh increased shoot biomass and, critically, shifted biomass allocation toward reproductive spikes (Zhang et al., 2025a). This warming-induced shift raised spike biomass and enhanced both the number and mass of seeds, while simultaneously prolonging seed floating duration and expanding the potential range of water-borne dispersal (Zhang et al., 2025a). The cascading effect on invasion is threefold: (i) elevated seed output increases propagule pressure on receiving shores; (ii) prolonged floating time extends the spatial reach of the invader; and (iii) reproduction skewed toward the late growing season alters the temporal window for competitive interactions with native germinants. A parallel study on *S. alterniflora* across three latitudinal common gardens (21°N, 28°N, 38°N) showed that cold stratification (4 °C for 0 or 4 months) promoted germination rate and index but reduced germination time, and that higher germination temperature amplified these effects from high- to low-latitude gardens (Wu et al., 2024). The interaction between cold stratification and warming varied among provenances: subtropical and temperate provenances benefited increasingly from cold stratification as temperature rose, whereas tropical provenances lost their germination advantage under warming (Wu et al., 2024). This reveals a biogeographic pattern, in which warm-adapted populations may migrate northward to secure adequate chilling while tropical lineages face contraction, and this pattern directly shapes the future range of the invader under climate change.

Collectively, these findings support a two-axis framework for global-change effects on invasion. Resource-subsidy drivers (elevated CO_2_, N deposition; Section 2.2) magnify the growth–defense trade-off in favor of resource-acquisitive invaders, as quantified in open-top chamber experiments (Zhang et al., 2024a; Zhao et al., 2022a). Disturbance-regime drivers (drought–rewetting, extreme temperature, warming amplitude) select for invaders with high phenotypic plasticity, rapid recovery, and flexible biomass allocation (Pan et al., 2026; Zhang et al., 2025a; Wu et al., 2024). The key distinction is that subsidy drivers operate continuously and favor growth investment, whereas disturbance drivers operate as pulses and favor resilience and plasticity. This dichotomy clarifies why some invaders thrive under gradual change while others surge specifically after extreme events, and it provides a mechanistic basis for the life-history stratification developed in Section 3.2.

Importantly, extreme climate does not affect all invaders uniformly. Annuals and perennials differ in their response to drought–rewetting: annuals capitalize on above-ground growth surges upon rewetting, while perennials leverage deeper root storage to buffer drought but show weaker rewetting recovery (Pan et al., 2026). Invasive populations also differ from native populations in their allocation strategies under warming: the invasive population of *Plantago virginica* increased above-ground biomass under warming and N addition, whereas the native population invested more in below-ground biomass (Luo et al., 2020). These differential responses underscore that extreme climate effects are contingent on both life-history strategy and invasion status, a theme revisited in Sections 3.2 and 4.3.

### 2.4 Heavy metal pollution from stress source to elemental defense weapon

Heavy metal pollution is another major feature of the Anthropocene ecosystem, traditionally viewed as purely toxic stress to organisms (Ai et al., 2022; Chen et al., 2021; Hu et al., 2020). However, recent studies have uncovered a striking ecological phenomenon: for some metal-tolerant and metal-accumulating plants, particularly invasive species, heavy metals can be shifted from lethal toxicants to elemental defensive weapons against herbivores (Wang et al., 2022b; Chen et al., 2021; Zhang et al., 2019). This strategy cleverly integrates abiotic stress factors into the biological defense system, reconstructing the rules of interspecific interaction in polluted environments (Wang et al., 2024; Shi et al., 2023; Zhao et al., 2018).

Taking cadmium (Cd) pollution as an example, its ecological effects go far beyond inhibiting plant growth (Ai et al., 2022; Xiao et al., 2018). Under Cd stress, the invasive plant *A. philoxeroides* can accumulate high concentrations of Cd in its aboveground tissues, particularly in leaves (Wang et al., 2022b). This high Cd accumulation exhibits strong toxicity to the specialist natural enemy *Agasicles hygrophila*, preventing it from completing its life cycle after feeding on high-Cd leaves (Wang et al., 2022b). Confronted with such chemical armor, *A. hygrophila* adopt an adaptive host shift, migrating from high-Cd invasive plants to the native sessile joyweed, which presents lower Cd accumulation capacity and potentially weakened organic defense responses under stress (Wang et al., 2022b; Henriksen et al., 2018). Meanwhile, the defense of the invasive plant against the generalist pest tobacco cutworm presents a different mechanism (Wang et al., 2020a; Xiao et al., 2019). Research indicates that the main factors inhibiting the growth and development of tobacco cutworm larvae are organic secondary metabolites like flavonoids in the invasive plant’s leaves, not the Cd element itself (Wang et al., 2022b; Long et al., 2019). This divergence in defensive strategies shows that invasive plants deploy inorganic elements (Cd) against specialist natural enemies and organic compounds against generalist pests. It reflects sophisticated defense resource allocation under polluted conditions (Long et al., 2023; Li et al., 2022a; Chen et al., 2021). Its comprehensive ecological effect is the construction of a highly unfavorable “trap” for native plants in polluted habitats (Luo et al., 2020; Lu et al., 2019). The invasive plant effectively avoids control by specialist natural enemies through elemental defense while simultaneously suppressing generalist pests with strong constitutive or induced organic defenses (Wang et al., 2022b, 2020). In contrast, the native sessile joyweed encounters a dual predicament. It serves as an alternative host for the specialist natural enemies escaping toxic stress, while still sustaining feeding damage from generalist pests that inherently favor this native species (Wang et al., 2022b; Henriksen et al., 2018). Heavy metal pollution markedly intensifies the apparent competition effect, a potent indirect competitive suppression mediated by shared natural enemies following stress-induced host switching (Zhang et al., 2023a; Luo et al., 2020; Lu et al., 2019).

Therefore, soil pollution inadvertently creates an asymmetric competitive advantage for invasive plants possessing elemental defense capabilities, altering the traditional invasion models based on resource competition or enemy release (Luo et al., 2020; Lu et al., 2019). This discovery has profound implications for the ecological management of polluted environments (Zhang et al., 2023a; Henriksen et al., 2018). It implies that in heavy metal-polluted areas, traditional biological control (e.g., releasing specialist natural enemies) may face failure or even generate negative ecological risks, as the natural enemy may attack non-target native plants (Zhang et al., 2023a; Henriksen et al., 2018). Simultaneously, this indicates that simple removal of invasive plants without concomitant soil remediation yields limited efficacy, since contaminated soil consistently confers defensive superiority on metal-tolerant invasive species (Ai et al., 2022; Hu et al., 2020). Understanding how heavy metal pollution evolves from a stress source to a “weapon” for invasive plants is the cornerstone for developing intelligent ecological management strategies for polluted habitats (Wang et al., 2022b; Chen et al., 2021). Furthermore, other heavy metals like zinc, nickel may also be used for defense by certain hyperaccumulator plants, and their ecological effects warrant further investigation (Sirakov et al., 2021; Shahzad et al., 2021). Heavy metal pollution may also indirectly affect plant competition patterns by modifying the structure and function of soil microbial communities, thus regulating plant-soil feedback (Shi et al., 2023; Wang et al., 2021; Zhao et al., 2018). Therefore, comprehensive assessment of heavy metal pollution ecological risks should extend beyond direct toxic impacts on individual plants, and elucidate how it regulates interspecific interaction networks, in turn shaping community structure and ecosystem functioning (Wang et al., 2024, 2022; Chen et al., 2021).

### 2.5 Geographic variation in plant traits and climate adaptation programming

Faced with heterogeneous environmental conditions across vast geographic spaces, plant populations are not homogeneous entities but form geographic variation patterns in traits that match climatic gradients through phenotypic plasticity and local adaptive evolution (Cao et al., 2024; Zhou et al., 2022). This variation constitutes a crucial adaptive strategy enabling plants to withstand environmental selection and facilitate colonization and range expansion (Zhou et al., 2022; Luo et al., 2020). A large-scale analysis of seed traits across 26 populations of the globally invasive weed *Ambrosia artemisiifolia* spanning 23 degrees of latitude and 20 degrees of longitude in China provides a classic example for understanding this climate adaptation regulation (Zhou et al., 2021). The study systematically measured over 20 traits including seed morphology, plant hormones, nutrients, and germination characteristics, finding significant and regular systematic variation along latitude/longitude gradients (Zhang et al., 2020a; Li et al., 2020). As latitude increases (corresponding to lower temperature), seed germination time is significantly delayed, while germination rate shows a decreasing trend (Zhou et al., 2021). Accompanying this are increases in seed size, the level of the germination-inhibiting hormone abscisic acid (ABA), and total fatty acid content serving as storage substances along rising latitudes (Zhou et al., 2021). Statistical analysis indicates that temperature is the dominant climatic factor driving this geographic variation in traits (Zhou et al., 2021). This series of co-varying traits outlines a clear map of adaptive strategies (Cao et al., 2024; Zhou et al., 2022): In warm, low-latitude regions with long growing seasons and relatively predictable environments, ragweed populations tend to adopt a “fast-investment, high-turnover” strategy (Zhang et al., 2020a; Li et al., 2020). They produce more numerous smaller seeds with lower ABA content and fast germination ability, striving to rapidly occupy ecological niches and complete their life cycle by leveraging the prolonged growing season (Zhou et al., 2021; Zhang et al., 2020a). Conversely, in cold, high-latitude regions with short growing seasons and potential risks like early frost, ragweed populations shift to a “conservative-investment, high-security” strategy (Zhang et al., 2020a; Li et al., 2020). They produce fewer but larger, resource-richer (high fatty acid) seeds and induce deeper or more flexible dormancy by increasing ABA content to delay germination, avoiding early spring uncertainties, and ensuring germinated seedlings have ample resource reserves to cope with stress and improve establishment success (Zhou et al., 2021; Zhang et al., 2020a). This trait modulation along environmental gradients profoundly reveals the microscopic mechanisms of invasion success (Zhou et al., 2022; Lu et al., 2019).

Invasion is not mere spatial expansion, but an adaptive radiation process characterized by rapid differentiation of population traits in response to local selection pressures (Cao et al., 2024; Zhou et al., 2022). Such phenotypic variation across geographic scale allows invasive plants to achieve precise adaptation to key climatic factors (e.g., temperature) in their new habitats, facilitating their successful colonization over extensive areas spanning from south to north (Zhou et al., 2021; Zhang et al., 2020a). Understanding this trait-climate association is crucial for predicting the dynamics of invasive species under future climate change (Zhang et al., 2023c, 2022). For example, based on current trait-temperature relationships, one can predict that under warming scenarios, high-latitude populations may evolve toward “smaller size and faster germination”, potentially altering their expansion speed and pattern (Zhou et al., 2021; Zhang et al., 2020a). Integrating such functional trait data into species distribution models or dynamic vegetation models can significantly enhance the mechanistic nature and accuracy of predictions, allowing us not only to predict changes in species’ distribution ranges but also to infer shifts in their adaptation strategies (Zhang et al., 2023c, 2022).

Therefore, the study of geographic variation in plant traits serves as a bridge connecting macroscopic biogeographic patterns with microscopic adaptation mechanisms and is core to understanding species responses to global change and predicting their future fate (Song et al., 2024; Cao et al., 2024; Zhou et al., 2022; Li et al., 2022b). Beyond seed traits, other functional traits including specific leaf area, photosynthetic characteristics, water use efficiency, and secondary metabolite composition also generally present geographic variation, together constituting a comprehensive adaptation strategy for plants to cope with environmental heterogeneity (Xiao et al., 2024; Li et al., 2020; Xiao et al., 2020). Future research needs to integrate genomics, transcriptomics, and phenomics to reveal the genetic and molecular regulatory basis underlying geographic trait variation and the relative contributions of phenotypic plasticity and local adaptation (Li et al., 2025c, Song et al., 2023; Yu et al., 2023).

## 3 Plant defense network responses to biotic stress

In the challenging natural environment, plants continuously face threats from various biotic stresses such as herbivorous insects and pathogenic microorganisms. To address these threats, plants have evolved a complex, precise, and highly intelligent defense system. This system is not a rigid “stimulus-response” device but a networked “immune system” capable of dynamic signal integration, priority decision-making, and optimal resource configuration based on the nature, intensity, timing, and combination of stresses. Its intelligence lies in its ability, under the fundamental constraint of limited resources, to weigh the costs and benefits of different defense strategies through crosstalk between core defense signaling pathways, ultimately outputting a defense response most conducive to maintaining its overall fitness (**Figure 2**). Profound analysis of regulatory logic and trade-off mechanisms of this defense network is essential for interpreting plant survival strategies, interspecific interactions, and even community assembly rules.

**Figure 2:**
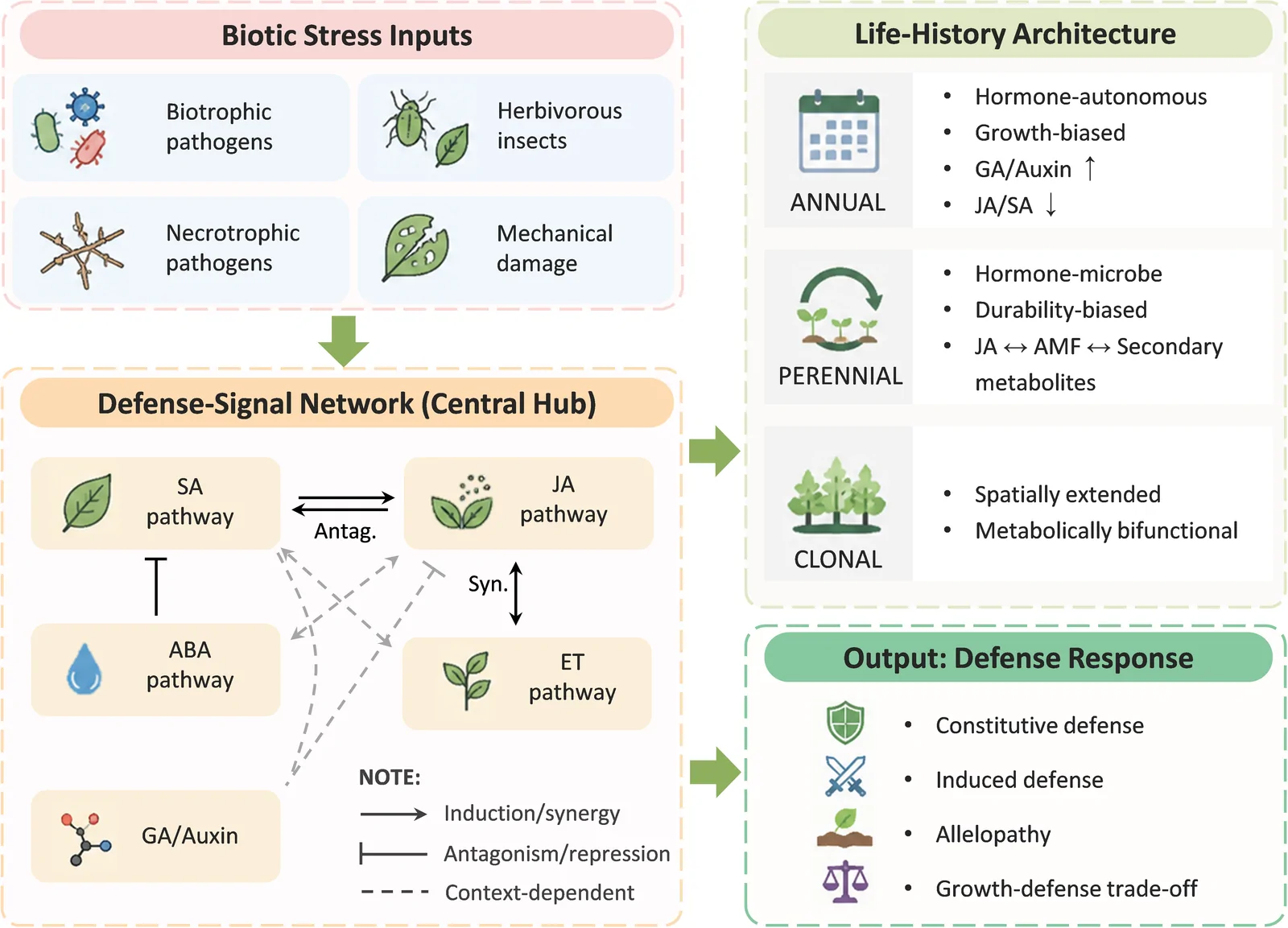
Conceptual framework of plant defense network responses to biotic stress. Biotic stress inputs include biotrophic pathogens, necrotrophic pathogens, herbivorous insects, and mechanical damage, which trigger a central defense-signaling network formed by crosstalk among SA, JA, ABA, ET and GA/Auxin pathways. The outputs of this signaling network diverge depending on plant life-history architecture, including annual, perennial, and clonal strategies. Annual plants exhibit hormone-autonomous, growth-biased regulation with elevated GA/Auxin and reduced JA/SA signals; perennials rely on hormone–microbe interactions and durability-oriented defense linked to JA–AMF–secondary metabolite signaling; clonal plants feature spatially extended and metabolically bifunctional traits. Ultimately, these divergent pathways generate multiple defense phenotypes, including constitutive defense, induced defense, allelopathy, and the growth–defense trade-off. Symbol explanations: “↑” indicated up-regulated; “↓” indicated down-regulated; “↔” indicated interaction; SA represented salicylic acid; JA represented jasmonic acid; ET represented ethylene; ABA represented abscisic acid; GA represented gibberellin; AMF represented arbuscular mycorrhizal fungi; Antag., antagonism; Syn., synergy.

### 3.1 Crosstalk and regulation in defense signaling pathways

The plant defense system is a highly integrated and intelligent network capable of prioritizing decisions and optimizing resource allocation through crosstalk between core signaling pathways based on the nature, intensity, and combination of stresses, rather than responding equally to all threats (Zhai et al., 2022; Yuan et al., 2021; Wang et al., 2020c). These regulatory decisions are fundamentally governed by dynamic antagonistic and synergistic interactions between the two key defense signaling pathways of salicylic acid (SA) and jasmonic acid (JA) (Song et al., 2021; Zhu et al., 2020). The SA pathway typically dominates defense against biotrophic pathogens (e.g., *Blumeria graminis*, *Pseudomonas syringae*), while the JA pathway mainly mediates responses to necrotrophic pathogens, herbivorous insects, and mechanical damage (Xiong et al., 2022; Xing et al., 2019). When plants face a single stress, the corresponding pathway is specifically activated (Sun et al., 2022; Zhu et al., 2020). However, in natural environments, plants more often encounter multiple, sequential, or simultaneous biotic stresses (Sun et al., 2022; Dong et al., 2021a). At this point, plants initiate precise regulatory decision internally (Sun et al., 2022). For example, when wheat faces simultaneous infection by the SA-pathway-inducing fungus *Blumeria graminis* and the JA-pathway-inducing fungus *Fusarium graminearum*, its defense response is not a simple superposition of the two pathways (Fu et al., 2025; Xiong et al., 2024). Research indicates that the SA signaling pathway frequently dominates in such scenarios, and its activation can suppress responses of the JA pathway to a certain extent (Yuan et al., 2021; Wang et al., 2020c). This apparent trade-off strategy essentially reflects the survival wisdom of plants under resource-limited conditions (Liu et al., 2024; Wang et al., 2023a). The synthesis of defensive compounds and signal transduction consume substantial carbon, nitrogen, and energy resources (Liu et al., 2024; Wang et al., 2023a). When multiple threats arrive simultaneously, plants must assess the relative threat level of different stresses to their fitness and allocate limited defense resources to the most urgent or lethal threat (Sun et al., 2022; Dong et al., 2021a). The antagonism between SA and JA pathways is likely the manifestation of this resource allocation decision at the molecular level (Song et al., 2021; Zhang et al., 2020b). This decision logic possesses high ecological rationality (Cao et al., 2024; Zhou et al., 2022). Through this intelligent signal integration and trade-off, plants can avoid exhausting resources due to over-defense, ensuring the maintenance of an optimal balance between growth, reproduction, and defense in complex and variable biotic stress environments, thereby maximizing fitness (Liu et al., 2024; Wang et al., 2023a). Understanding the regulation rules of this defense decision-making is of fundamental significance for breeding crops with broad-spectrum resistance, predicting plant performance under complex pest and disease pressures, and understanding the co-evolutionary relationships between plants and multiple natural enemies in natural communities (Song et al., 2023; Yu et al., 2023).

However, the rigid SA-JA antagonistic trade-off summarized from model crops cannot fully explain the unique adaptive strategies of invasive plants. Recent comparative molecular experiments paired invasive taxa with their native congeners have verified that invasive species evolve plastic, stress-dependent rewiring of SA/JA crosstalk to gain competitive superiority under compound biotic stresses. Under fungal pathogen infection, the invasive weed *Alternanthera philoxeroides* exhibits far stronger SA-mediated repression of JA cascades than its native congener; pathogen-triggered upregulation of SA biosynthetic genes restrains JA defensive transcripts and slows tissue necrosis, conferring enhanced disease resistance (Manoharan et al., 2019). When challenged by mixed chewing lace bugs and sap-feeding aphids, the exotic herb *Solidago altissima* weakens canonical SA-JA mutual inhibition and redistributes nitrogen to underground organs for compensatory growth, a trait absent in sympatric native herbs (Kollberg et al., 2026). During interspecific competition with native vegetation, the invasive plant *Flaveria bidentis* simultaneously downregulates both SA and JA defense programs while activating growth-related auxin and gibberellin signaling to accelerate biomass accumulation and resource pre-emption (Zhang et al., 2025b). Epiphytic symbiotic fungi drive persistent SA accumulation in the invasive liana *Ipomoea cairica*, which suppresses JA-dependent anti-insect metabolism and prioritizes pathogen tolerance in humid habitats (Xu et al., 2024a). The creeping invader *Sphagneticola trilobata* further displays ontogenetic JA trade-offs: it limits JA production during vegetative expansion to invest in allelochemicals inhibiting native seedlings, and only upregulates JA pathways at flowering to safeguard reproductive tissues (Hu et al., 2026). Collectively, unlike the static SA-JA antagonism observed in model crops, invasive plants remodel hormone signaling networks contextually under diverse superimposed stresses, and this flexible molecular regulation constitutes a core molecular foundation supporting their invasion success.

Besides SA and JA, other hormones such as ethylene, abscisic acid, and gibberellins are also involved in the cross-regulation of defense signals, forming a more complex signaling network (Li et al., 2025b; Gupta et al., 2024). Furthermore, global change factors such as warming and elevated CO_2_ concentration may alter the balance point of this defense decision by affecting hormone synthesis or signal transduction, thereby impacting plant disease and pest resistance in changing environments (Luo et al., 2020; Yu et al., 2019; Wu and Ding, 2019). Therefore, future research should thoroughly dissect the molecular mechanisms and ecological consequences underlying the integration and decision-making of plant defense signaling networks under multiple stresses and global change scenarios (Bawa et al., 2024; Song et al., 2023; Yu et al., 2023).

### 3.2 Differences in defense signal network architecture of invasive plants in life history strategies

The preceding sections treated invasive plants as a functionally homogeneous group in terms of defense signaling. This simplification obscures a central axis of variation: the architecture of the SA–JA–ET–ABA defense network is itself shaped by life-history strategy, and the same global-change drivers elicit qualitatively different hormonal responses in annual, perennial, and clonal invaders (Zhang et al., 2020c). In this sections that follow, we therefore describe three idealized architectural types: annual, perennial, and clonal, and then link each type to the global-change drivers that differentially amplify or suppress its characteristic defense strategy.

Annual invasive plants operate under a compressed temporal window between establishment and reproduction, which selects for a defense network biased toward rapid resource reallocation rather than durable defense investment. When *Flaveria bidentis* is grown in competition with native congeners, auxin (IAA) and gibberellin (GA_3_) levels rise significantly, accompanied by up-regulated *ARF* and *GID1* expression. Jasmonic acid and salicylic acid, together with their signaling genes *COI1*and *TGA*, are concurrently down-regulated, removing the antagonistic constraint imposed by defense hormones on growth hormones (Zhang et al., 2025b). This hormone-mediated growth–defense decoupling is not unique to *F. bidentis*. *Ambrosia trifida*, a globally invasive annual plant, deploys a chemically differentiated regulatory network comprising four secondary metabolites (vanillin, umbelliferone, trifolin, angelicin). This network integrates legacy environmental cues derived from senesced vegetation and real-time seed bank signals, thereby priming seed rapid establishment capacity via reprogramming of ribosome biogenesis and hormone signaling pathways (Chen et al., 2026). The hormonal signature of annual invaders under competition therefore converges on a consistent pattern: GA/auxin up-regulation coupled to JA/SA down-regulation, with the freed metabolic budget redirected to height and biomass accumulation. *Eupatorium adenophorum* further exemplifies this stress-adaptive trade-off. Under mechanical wounding and generalist herbivory, this invasive species exhibits weaker constitutive induction of JA and JA-Ile defenses than its native congener *E. heterophyllum*, yet compensates via substantially enhanced biomass regrowth (Wang et al., 2023c). Annual invaders thus resolve the growth-defense trade-off in favor of growth, leaning on compensatory capacity rather than on defense intensity.

Perennial invasive plants, by contrast, couple the SA–JA network tightly to long-term secondary metabolite accumulation and to mutualistic soil microbes, producing a defense architecture that is slower to reconfigure but more durable. In the perennial *Sphagneticola trilobata*, JA and arbuscular mycorrhizal fungi (AMF) work synergistically to improve herbicide resistance. Glyphosate exposure promotes AMF colonization and alters the composition of rhizosphere AMF communities; elevated AMF colonization further increases endogenous JA accumulation and abundance of stress-related metabolites. When JA and AMF function together, they generate a strong synergistic effect that simultaneously enhances plant growth and glyphosate tolerance (Xiong et al., 2025; Guo et al., 2024b). This indicates that SA–JA signaling network in *S. trilobata* does not operate independently. Instead, it is tightly integrated into a tripartite regulatory axis involving JA, AMF and secondary metabolism. As such, the plant’s ability to withstand environmental disturbance relies not only on endogenous hormonal regulation but also on its association with rhizosphere microbial symbionts. A similar microbe-dependent invasion strategy has been documented in *Solidago canadensis*. In this species, AMF-induced positive plant–soil feedback benefits invader growth and strengthens its competitive suppression of native plants, with negligible inhibitory effects on the invader itself (Guo et al., 2025; Dong et al., 2021b). Further evidence shows that the positive plant–soil interaction of *S. canadensisis* realized through directly or indirectly regulating phenotypic traits including plant height, leaf number and number of rhizomes, with invasive AMF significantly increasing belowground and total biomass of the invasive plant but not those of native species (Zhang et al., 2021b). Distinct architectural patterns can be distinguished: annuals possess hormone-autonomous defense networks biased toward growth, while perennials maintain hormone–microbe-integrated networks favoring long-term durability.

Clonal invasive plants exhibit a distinct structural strategy: their defense signaling networks span beyond individual ramet. In *S. trilobata*, local ABA application to one ramet alleviates oxidative stress in distal ramets through the stolon connection, raising proline and soluble protein in the third ramet and total biomass of the clone; the same ABA signal fails to produce equivalent systemic benefit in the native congener *S. calendulacea* (Wang et al., 2025c). Clonal integration thus permits non-resource stress signals to propagate across ramets, effectively turning the clone into a distributed defense network in which local cue perception protects distal modules. *Alternanthera philoxeroides* adds a metabolic dimension: during expansion its stem nodes toggle between a “growth-oriented” metabolic profile (activation of phenylpropanoid biosynthesis, pentose phosphate pathway, tyrosine metabolism) and a “survival-oriented” profile enriched in trans-cinnamic acid, skimming, ursolic acid, and inactive auxin (indole-3-acetyl-aspartate), a dynamic metabolic reprogramming between growth and defense modes that underlies its success across aquatic–terrestrial gradients (Li et al., 2025a). For clonal invaders, the SA–JA–ABA network is spatially extended and metabolically bifunctional, allowing defense investment to be allocated optimally across the clone rather than locked into a single ramet.

The foregoing descriptions treat life-history strategy as an intrinsic property of the invader, but the selective environment created by global change is what amplifies or suppresses each architectural type. Resource-subsidy drivers (elevated CO_2_, nitrogen deposition; Section 2.2) preferentially favor annual invaders whose defense network is already biased toward growth. The competitive advantage of the invasive population of *Plantago virginica* under combined warming and N addition arises from greater aboveground biomass allocation, whereas the native population invests biomass belowground; this illustrates how resource subsidies and warming together reinforce the annual “grow fast” strategy (Luo et al., 2020). Conversely, disturbance-regime drivers (drought–rewetting pulses, warming amplitude) select for invaders whose defense architecture is buffered by microbial partnerships or spatially distributed across clones. The documented enhancement of *S. trilobata*’s herbicide resistance by the JA–AMF axis (Xiong et al., 2025) and the clonal distribution of ABA-mediated stress protection in the same species (Wang et al., 2025c) are both expressions of this disturbance-resilience mode.

These three architectural types carry direct implications for how global change modulates invasion. Resource-subsidy drivers (elevated CO_2_, N deposition) amplify the growth-biased hormone network of annuals, as documented in Section 2.2 and illustrated by the divergent allocation strategies of invasive versus native *P. virginica* populations under combined warming and N (Luo et al., 2020). Disturbance-regime drivers (extreme drought–rewetting, warming amplitude) select for the microbially coupled resilience of perennials and the distributed defense of clonal invaders. This aligns with findings that invasive plants produce stronger chemical signaling via root exudates, including elevated quercetin and strigolactone concentrations, to foster associations with AMF and other beneficial soil microbes (Yu et al., 2022). A unified theory of invasion under global change therefore requires that the SA–JA–ET–ABA defense network be typed not only by its endogenous state but by the life-history strategy that shaped its architecture and by the global-change driver that amplifies it. Subsequent sections on interspecific interaction networks and management interventions must likewise be stratified by this signaling-architecture typology: hormone-targeted control that works against an annual invader’s GA/auxin bias will not translate to a perennial whose defense is microbe-mediated, nor to a clonal invader whose ABA signal is systemically distributed across ramets.

### 3.3 Diversity of chemical defense and its ecological functions

Throughout long-term evolution, plants have built a vast and diverse chemical defense “arsenal”, whose products are collectively termed secondary metabolites (Ma et al., 2023; Long et al., 2023). These compounds not only directly exert toxic or deterrent effects against insects and microbes but also play key roles in intra- and interspecific information exchange, constituting the chemical language of complex ecological interactions (Martin-Cacheda et al., 2025; Wang et al., 2020a). Direct defensive substances include alkaloids, terpenoids, phenolics (e.g., flavonoids, tannins), etc., which protect plant tissues by interfering with herbivore digestion, nervous systems, or through direct toxicity (Wagner et al., 2020; Long et al., 2019).

Beyond these direct actions, chemical defense extends to more fine-scale ecological functions (Gao et al., 2020). Allelopathy is one example, where plants release specific chemicals into the environment to inhibit seed germination, seedling growth, or nutrient absorption of neighboring competitors, thereby gaining an advantage in resource competition (Xiao et al., 2024; Feng et al., 2023). More interestingly, some plants can even adjust their allelopathic strategy based on the identity of the competitor (Ding et al., 2023). For instance, resistant barnyard grass *Echinochloa crusgalli* reduces the release of allelochemicals when coexisting with kin conspecifics, showing more “cooperative” or tolerant tendencies; whereas it significantly enhances allelopathic effects when competing with non-kin individuals, exhibiting a strong “competitive” stance (Ding et al., 2023; Yu et al., 2022). This chemical behavior adjustment based on kin recognition is a form of sophisticated ecological intelligence that helps optimize intraspecific resource use efficiency while enhancing suppression of interspecific competitors, maximizing the fitness of kin individuals (i.e., inclusive fitness) (Luo et al., 2020; Lu et al., 2019). Additionally, VOCs released by plants after herbivory can both attract natural enemies (indirect defense) and warn neighboring conspecific or heterospecific plants of potential threats, priming their defenses (Martin-Cacheda et al., 2025; Xiao et al., 2019). The diversity of chemical defense is also reflected in its inducibility (Liu et al., 2024). Many defensive compounds are not present at high concentrations continuously but are rapidly synthesized upon attack; this induced defense can conserve resources and maximize defense efficacy (Xing et al., 2024; Zhang et al., 2021c).

The composition and concentration of plant chemical defenses are regulated by multiple factors including genetics, environment (e.g., nutrient status, light, stress), and biotic interactions (e.g., microbial symbiosis) (Chen et al., 2021; Xiao et al., 2020). Global change factors such as warming, elevated CO_2_ concentration, and nitrogen deposition may regulate the plant’s chemical defense profile by affecting its physiological metabolism, thereby altering its ecological niche and interaction relationships within the community (Liu et al., 2022; Wu and Ding, 2019). Therefore, the diversity and plasticity of chemical defense act as core strategy for plants to cope with complex biotic environments, drive niche construction, and engage in interspecific interactions (Muhammad et al., 2025; Cao et al., 2024). In-depth research on the synthesis, regulation, and ecological functions of plant secondary metabolites is crucial for developing new plant protectants, understanding biodiversity maintenance mechanisms, and predicting community dynamics under global change (Yang et al., 2025; Xu et al., 2024b).

### 3.4 Growth-defense trade-offs and source-sink relationship regulation

Plant resources are limited. Investment in defense (e.g., synthesizing secondary metabolites, constructing physical barriers, activating defense signaling pathways) inevitably consumes resources that could otherwise be used for growth and reproduction. This inherent contradiction represents the classic growth-defense trade-off in ecology and evolutionary biology (Long et al., 2025; Qin et al., 2023a). This trade-off is not fixed but is dynamically regulated by the plant’s internal physiological state and external environmental conditions, with source-sink relationships playing a central role (Peng et al., 2018; Guo et al., 2018). The source primarily denotes organs that synthesize photosynthates and related precursor substances (e.g., mature leaves), while the sink refers to organs that consume or store photoassimilates (e.g., roots, shoot apices, flowers, fruits, seeds) (Ding et al., 2023; Bawa et al., 2022).

When plants suffer biotic stress, the activation of defense signals triggers the redistribution of photosynthates (Zhao et al., 2021). For example, more carbon skeletons may be directed toward the phenylpropanoid pathway to synthesize phenolic, flavonoid, and other defense compounds, thereby reducing the flow of assimilates to growth sinks (e.g., new leaves, roots) (Li et al., 2021; Gao et al., 2021). This resource reallocation is the physiological basis of the growth-defense trade-off (Li et al., 2023). Environmental resource availability profoundly influences this trade-off (Zhao et al., 2022b). Nitrogen and water are key limiting resources for plant growth and metabolism (Chong et al., 2022). Nitrogen addition typically alleviates plant nitrogen limitation and promotes growth, but its impact on defense investment is complex (Liu et al., 2023). On one hand, extra nitrogen may be allocated to synthesize nitrogen-containing defensive compounds, such as alkaloids and cyanogenic glycosides. On the other hand, the prioritization of growth demands may dilute defensive investment or alter the category of defensive compounds, switching from carbon-based defense to nitrogen-based defense) (Jiang et al., 2019; Zhao et al., 2018). Water status is equally important; drought stress may induce the synthesis of certain defense compounds but simultaneously severely limits overall photosynthetic production and resource availability (Xiao et al., 2025). Research indicates that the interaction of nitrogen and water can alter the nutrient limitation status of soil microorganisms, further affecting plant nutrient acquisition and growth-defense balance through plant-soil feedback (Zhang et al., 2024b; Long et al., 2020a). Furthermore, plant developmental stage and sink strength also regulate this trade-off (Cai et al., 2019). During periods of vigorous vegetative growth or seed filling (high sink strength), plants may tend to allocate more resources to growth and reproduction, with relatively lower defense investment; whereas under severe stress, defense is prioritized (Qin et al., 2023b; Wang et al., 2023b).

Understanding how resource availability (e.g., nitrogen deposition, altered precipitation patterns) under global change backgrounds modulate plant growth-defense trade-off strategies by regulating source-sink relationships and internal resource allocation is crucial for predicting plant population dynamics, community structure, and changes in co-evolutionary relationships between plants and herbivores (Guo et al., 2024a; Kang et al., 2019). This is not only a core issue in theoretical ecology but also has practical significance for balancing crop yield and resistance in agricultural management and for selecting plant materials with suitable traits in ecological restoration (Wang et al., 2022b; Liu et al., 2022). Future research needs to quantify the resource allocation ratio between growth and defense under different environmental conditions and reveal the underlying genetic and molecular regulatory mechanisms (Zhou et al., 2021).

## 4 Reconstruction of interspecific interaction networks

Physiological and trait adaptive regulation at the individual plant level does not occur in an ecological vacuum. On the contrary, these changes cascade upwards and reshape the entire ecosystem’s interspecific interaction network by altering the roles of plants as resource providers, information releasers, competitors, and symbiotic partners. Global change factors act like invisible “network engineer”, regulating the state and behavior of key nodes (plants) in the network, thereby changing the strength and nature of connections between nodes, and ultimately leading to the reconstruction of the entire ecological network structure. This reconstruction is reflected in the complex relationship dynamics between plants and insects, plants and microorganisms, and among plants themselves, emerging from microscopic changes in chemical communication to macroscopic changes in community composition and ecosystem function (**Figure 3**).

**Figure 3:**
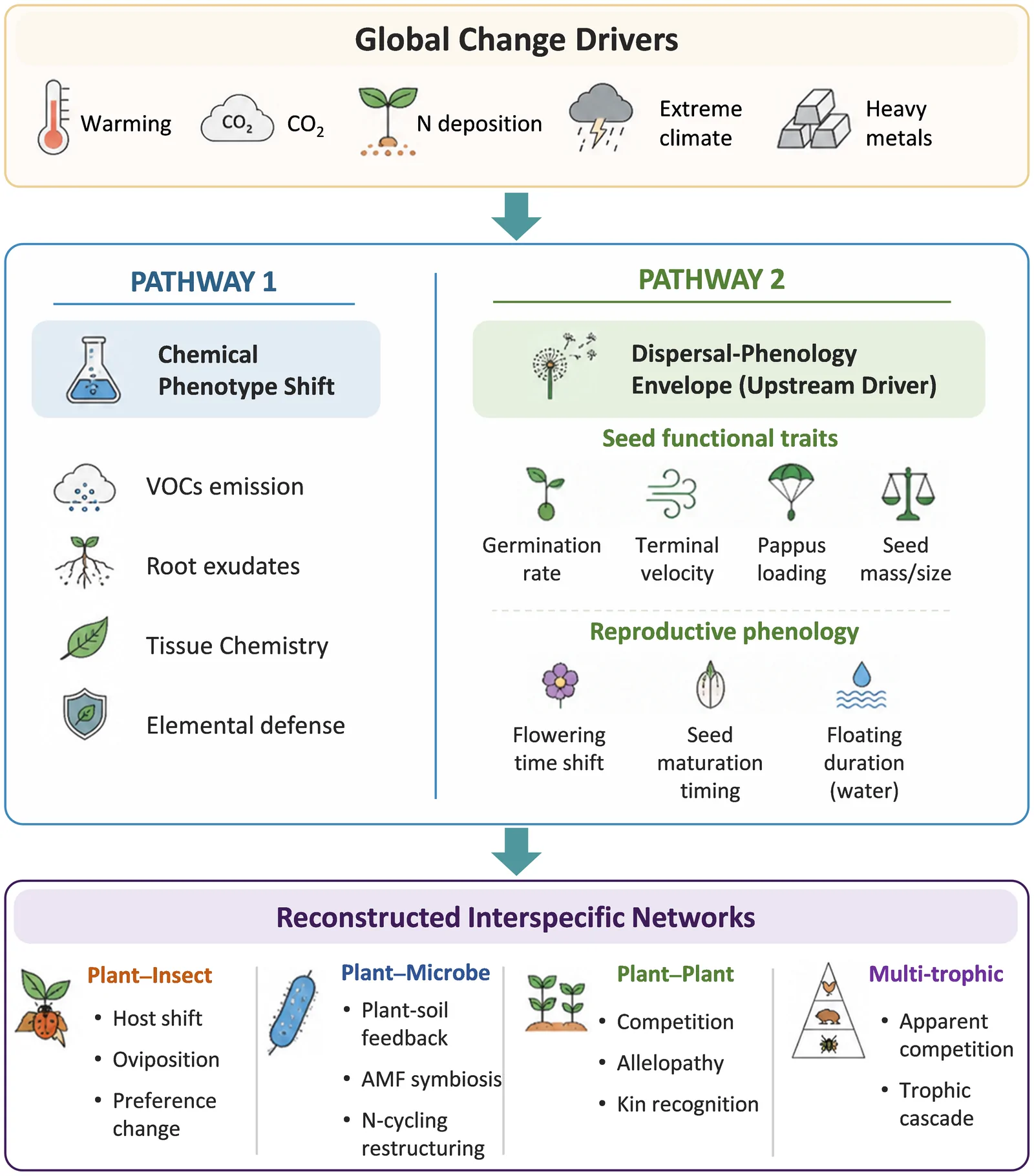
Conceptual framework for global-change-mediated reconstruction of interspecific interaction networks. Warming, elevated CO_2_, N deposition, extreme climate, and heavy metals act through two pathways: chemical phenotype shifts (VOCs, root exudates, tissue chemistry and elemental defense) and the dispersal–phenology envelope (seed functional traits and reproductive phenology). These processes jointly restructure plant–insect, plant–microbe, plant–plant and multi-trophic interaction networks, altering outcomes such as host preference, plant–soil feedback, allelopathy and trophic cascades. N represented nitrogen; VOCs represented volatile organic compounds; AMF represented arbuscular mycorrhizal fungi.

### 4.1 Modulation of plant-insect interactions

Global change factors profoundly reshape plant-insect interaction networks via modulating plant physiological and chemical phenotypes (Liu et al., 2022; Luo et al., 2020). Such modulation is highly context-dependent and species-specific, with the ultimate ecological effect depending on the nature of plant chemical changes, the degree of dietary specialization of the insect, and the plasticity of its sensory and physiological systems (Wang et al., 2020a; Xiao et al., 2019). Warming may alter the composition of plant volatile organic compounds, thereby affecting long-distance host location by insects (Martin-Cacheda et al., 2025; Liu et al., 2022). Heavy metal pollution may transform plant tissues into a toxic medium for sensitive insects, forcing specialist insects to shift hosts (Wang et al., 2022b; Henriksen et al., 2018). Elevated atmospheric CO_2_ concentration may alter the carbon-to-nitrogen ratio of plant tissues, affecting their nutritional quality and defensive compound concentration, thereby changing the development and population dynamics of herbivores (Zheng et al., 2019; Wu and Ding, 2019).

These changes do not occur in isolation but act together on complex food webs (Wang et al., 2024). For example, warming-induced plant chemical changes may decrease a plant’s attractiveness to a generalist pest but simultaneously enhance or diminish its attractiveness to a specific specialist natural enemy (Liu et al., 2022; Henriksen et al., 2018). Heavy metal pollution, while forcing specialist natural enemies to shift hosts, may not affect the preference of generalist pests, leading to disproportionate pressure on native plants (Wang et al., 2022b; Henriksen et al., 2018). This asymmetric regulation of interactions alters the distribution of herbivory pressure borne by various plant species within the community, thereby affecting interspecific competitive balance (Luo et al., 2020; Lu et al., 2019). In some cases, this may weaken the competitive ability of native plants, creating windows of opportunity for invasive plants; in other cases, it may alter natural enemy-pest dynamics, affecting the efficacy of biological control (Zhang et al., 2023a; Lu et al., 2019). Therefore, understanding the rules of plant-insect interaction modulation under global change is key to predicting future pest outbreak trends in agricultural ecosystems, assessing the stability and succession direction of natural ecosystems, and formulating adaptive management strategies (Luo et al., 2020; Wu and Ding, 2019). This requires that in research we not only focus on single stress factors but also consider the compound effects of multiple global change drivers and assess their ecological consequences within a multi-trophic network framework (Wang et al., 2024; Xing et al., 2024).

### 4.2 Regulation of plant-soil feedbacks

Plants interact not only with insects and competitors aboveground but also engage in continuous and close dialogue with vast soil microbial communities underground through plant-soil feedback, a process that is a key link connecting individual plants to ecosystem function (Feng et al., 2023; Bakker et al., 2020). Plants profoundly shape the physical, chemical, and biological properties of the rhizosphere and surrounding soil through litter input and root exudates (including sugars, amino acids, organic acids, secondary metabolites, etc.) (Yu et al., 2022; Zhao et al., 2021). Different plant species or genotypes produce distinct litter and exudates, thereby cultivating unique soil microbial communities (including bacteria, fungi, protists, nematodes, etc.) (Shi et al., 2023; Zhao et al., 2018). These microbial communities, in turn, influence plants through various pathways: beneficial microorganisms (e.g., mycorrhizal fungi, nitrogen-fixing bacteria, biocontrol agents) can promote plant nutrient uptake and enhance stress resistance; whereas pathogenic microorganisms inhibit plant growth (Wang et al., 2021; Bakker et al., 2020). Combining root exudate metabolomics and soil metagenomics builds a molecular framework to decipher how invasive plants secrete allelochemicals to reshape rhizosphere microbes and form self-beneficial plant-soil feedbacks (Qi et al., 2023; Mishra et al., 2022). These unique root compounds enrich soil pathogens or break native mycorrhizal symbioses, securing long-term competitive dominance over local flora (Mishra et al., 2022). Integrating multi-layer molecular omics and community meta-omics further connects small-molecule root signals to ecosystem-level soil alterations, repairing the logical gap between micro molecular regulation and macro ecological phenomena (Zhan, 2025). Therefore, by altering the soil environment, plants affect the performance of conspecific or heterospecific plants subsequently growing in the same patch. This is plant-soil feedback, which can have positive (benefiting itself or kin) or negative (inhibiting itself) effects (Feng et al., 2023; Yu et al., 2022).

Global change factors, including nitrogen deposition, shifted precipitation regimes, and climate warming, modulate the structure and function of soil microbial communities. These factors modify plant primary productivity and metabolic processes, thereby changing the quantity and quality of litter inputs and reshaping the composition of root exudates (Xiao et al., 2025; Luo et al., 2020). For example, nitrogen addition may alleviate nitrogen limitation for soil microorganisms, alter the carbon-nitrogen metabolism of microbial communities, affect litter decomposition and nutrient mineralization rates, and subsequently influence plant nutrient acquisition strategies through feedback (Li et al., 2023, 2021). Water changes similarly affect soil microbial activity and community composition, thereby modulating the strength and direction of plant-soil feedback (Xiao et al., 2025; Zhang et al., 2023b). Invasive plants often can establish positive feedback by creating soil microbial communities favorable to themselves (e.g., recruiting specific symbiotic bacteria or suppressing soil-borne pathogens), thereby promoting their invasion success (Feng et al., 2023; Yu et al., 2022).

Understanding how global change, by modulating plant belowground inputs, regulates the plant-soil feedback loop is crucial for predicting ecosystem carbon and nitrogen cycling, plant community succession, and the expansion potential of invasive plants (Long et al., 2020b; Luo et al., 2020). Modulating soil microbial communities, for example via inoculation with beneficial microorganisms or regulation of soil environment, has emerged as a promising strategy in ecological restoration and sustainable agriculture to improve ecosystem functions and plant health (Yang et al., 2025; Long et al., 2020b; Luo et al., 2020; Bakker et al., 2020).

### 4.3 Ecological strategies of invasive plants and interspecific competition

The success of invasive plants in new habitats often relies not on a single advantage but on the synergistic effect of multiple ecological strategies that give them an upper hand in interspecific competition (Cao et al., 2024; Zhou et al., 2022). Firstly, many invasive plants exhibit extremely high phenotypic plasticity and rapid evolutionary ability, enabling their key traits (e.g., phenology, biomass allocation, defense characteristics) to rapidly adapt to local environmental conditions, as shown by the systematic variation of ragweed seed traits along a latitudinal gradient (Zhou et al., 2021; Zhang et al., 2020a). Secondly, the “enemy release” hypothesis posits that invasive plants, leaving their native range, escape control by their specialist natural enemies, allowing them to invest more resources in growth and competition (Zhang et al., 2023a; Henriksen et al., 2018). However, they may also encounter new generalist natural enemies in the new environment, where strong chemical defenses (e.g., flavonoids) or unique defense strategies (e.g., elemental defense) become crucial (Long et al., 2023; Wang et al., 2022b). Thirdly, allelopathy serves as a powerful competitive tool for numerous invasive plants, which restrain the growth of adjacent native species via chemical secretion (Ding et al., 2023; Feng et al., 2023). More complexly, certain invasive species such as resistant barnyard grass can flexibly modulate allelopathic intensity according to the kinship of competing plants, enhancing allelopathic output when facing non-kin competitors to maximize competitive gain (Ding et al., 2023; Yu et al., 2022). Additionally, invasive plants may create favorable “soil feedback” for themselves by altering soil microbial communities (Feng et al., 2023; Yu et al., 2022). For example, they may recruit specific symbiotic bacteria to promote their own nutrient uptake or inhibit beneficial bacteria or promote pathogens associated with native plants through root exudates, thereby indirectly suppressing competitors (Yu et al., 2022; Wang et al., 2021). In terms of resource utilization strategy, invasive plants often have higher resource use efficiency (e.g., for light, water, nutrients) or can acquire and utilize resources more quickly during resource fluctuations (Luo et al., 2020; Lu et al., 2019). When facing global changes like warming or pollution, invasive plants may exhibit stronger adaptability or ability to exploit new opportunities than native plants, such as utilizing warming to alter insect preferences or using pollution for elemental defense (Wang et al., 2022b; Liu et al., 2022).

Underlying these multidimensional strategies is a common mechanistic hinge: seed functional traits and reproductive phenology operate as the upstream drivers that determine which species arrive at a site, when they arrive, and in what density, thereby pre-configuring the structure of interspecific networks before chemical signaling or microbial mediation begins. The empirical basis for this hinge is now well established. Across nine *Asteraceae* species sampled along roadsides in the Ailao Mountain region, invasive species attained significantly higher local plant density than natives, and this density advantage tracked seed germination rate: species density was significantly positively correlated with germination rate across all tested *Asteraceae*, while species frequency showed no significant correlation with any measured seed trait (Huang et al., 2025). The germination–sedimentation trade-off that constrains natives was decoupled in invaders. Among natives, germination rate was negatively correlated with sedimentation velocity, whereas invasive *Asteraceae* exhibited no such trade-off, enabling high germination to coexist with effective dispersal potential (Huang et al., 2025). At the within-species scale, *Chromolaena odorata* illustrates how invasion reshapes this envelope: introduced Asian populations produced higher seed numbers per capitulum, lighter seeds, and seeds with lower terminal velocity (higher potential dispersal ability) than native American populations, while the native range exhibited a seed-number–seed-mass trade-off absent in the introduced range (Li et al., 2024). Along climatic gradients, seed-trait strategies shift systematically: across 26 populations of *Ambrosia artemisiifolia* spanning 23° latitude and 20° longitude in China, germination time was significantly delayed with increasing latitude, germination rate showed the reversed pattern, and seed size, abscisic acid, and fatty acid content all increased from low to high latitude, with temperature identified as the dominant driver of this variation (Zhou et al., 2021). Extreme climate acts directly on this envelope: in *Spartina alterniflora*, a +1.5 °C growing-season warming significantly increased shoot biomass, shifted biomass allocation toward spikes, enhanced seed production in both number and mass, and prolonged seed floating time, collectively amplifying both clonal expansion and sexual dispersal (Zhang et al., 2025a). Collectively, these patterns define what we term the dispersal-phenology envelope of plant invasion: seed functional traits (germination rate, seed mass, pappus loading, terminal velocity) set the local dispersal kernel and determine propagule arrival sequence and density, while climate-driven shifts in reproductive phenology (flowering time, seed-allocation patterns, floating duration) alter the temporal and spatial window of species interactions. This envelope is the quantifiable variable that translates global-change drivers into interspecific network reconstruction. This mechanistic link must be explicitly incorporate into species distribution models to avoid systematic over- or underestimation of invasion spread.

The combined application of these strategies enables invasive plants to establish advantages across multiple dimensions: resource competition, enemy defense, and environmental impact, thereby displacing native species in new habitats, altering community structure and ecosystem function (Cao et al., 2024; Zhou et al., 2022). Understanding these multi-dimensional invasion strategies is the basis for developing effective prevention, control, and management measures (Yang et al., 2025; Zhang et al., 2023a).

### 4.4 Reproductive and dispersal traits as drivers of network reconstruction under global change

The preceding subsections documented how global-change drivers reshape plant-mediated interspecific interactions through chemical phenotypes and microbial partnerships. Yet these interaction networks are themselves anchored by the reproductive and dispersal traits of the interacting plants: a species cannot enter a competitive, trophic, or facilitative relationship it never reaches. We argue that seed functional traits and globally induced shifts in reproductive phenology act as upstream drivers of community assembly. These factors determine the arrival timing, density, and identity of colonizing species, thereby preconfiguring interspecific network structure prior to chemical and microbial modulation (Zhou et al., 2021).

#### 4.4.1 Direction 1: seed functional traits as predictors of local dispersal outcome

Across nine Asteraceae species (five invasive, four native) sampled along roadsides in the Ailao Mountain region, invasive species attained significantly higher local plant density than natives, and this density advantage tracked seed germination rate: species density was significantly positively correlated with germination rate across all tested Asteraceae, while species frequency showed no significant correlation with any measured seed trait (Huang et al., 2025). Distinct differences in the germination–sedimentation trade-off exist between invasive and native plant species. Native species exhibited a negative correlation between germination rate and sedimentation velocity, representing a classic seed ecological trade-off that limits local population expansion. Invasive Asteraceae species, by contrast, have decoupled this constraint: their germination performance is independent of sedimentation velocity, enabling simultaneous high germination capacity and strong dispersal potential (Huang et al., 2025). Accordingly, seed morphological characteristics, including sedimentation velocity, pappus loading, and seed length and width, cannot fully predict realistic dispersal outcomes (Wang et al., 2022a). Instead, the coupling or decoupling of germination competence and dispersal ability fundamentally differentiates the seed strategies of invasive and native plants.

This within-species lens gains sharper resolution when the same species is compared across its native and introduced ranges. In *Chromolaena odorata*, introduced Asian populations produce higher seed numbers per capitulum, lighter seeds, and seeds with lower terminal velocity (greater potential dispersal ability) than native American populations, alongside divergent trait-covariation architecture: the seed-number–seed-mass trade-off present in native-range populations is absent in the introduced range (Li et al., 2024). Latitudinal and longitudinal trends of seed traits further indicate that invasive plants deploy adaptive strategies along climatic gradients, with seed germination time, seed size, and hormone profiles varying systematically with latitude and temperature (Zhou et al., 2021). A classic demonstration comes from *Triadica sebifera*, where introduced populations differ significantly from native populations in seed properties and germination behavior, confirming that seed-trait divergence between ranges is a general mechanism underlying invasion success (Zhang et al., 2020a). The implications for network reconstruction are direct. Lighter, faster-germinating, and more widely dispersed seeds increase propagule pressure at the invasion leading edge. This process strengthens competition with resident natives, modulates herbivore host-range overlap, and reorganizes species arrival sequences, which ultimately govern the assembly of facilitative and antagonistic interactions during community establishment. Seed functional traits are thus not passive descriptors; they are the quantitative variables that predict local dispersal kernels and, consequently, the topology of the emerging interspecific network.

#### 4.4.2 Direction 2: extreme climate reconfigures networks by shifting reproductive phenology and seed-dispersal traits

Global-change drivers do not leave reproductive traits static. In *Spartina alterniflora*, two years of experimental growing-season warming (+1.5 °C) markedly increased shoot biomass and, importantly, shifted plant biomass allocation toward reproductive spikes. This warming-induced shift promoted greater spike biomass and enhanced seed output in both quantity and mass. Warming additionally prolonged seed floating duration, further expanding the potential range of water-mediated dispersal (Zhang et al., 2025a). The cascading effect on network structure is threefold: (i) elevated seed output increases propagule pressure on receiving shores; (ii) prolonged floating time expands the spatial reach of the invader, bringing it into contact with previously unreached native assemblages; (iii) phenological shifts, whereby reproduction becomes biased toward the late growing season, alter the temporal window for competitive interactions between invader seedlings and native germinants. The management implication derived directly from this mechanism is that cutting of invasive plants at an early flowering stage disrupts the warming-driven increase in seed production and floating dispersal (Zhang et al., 2025a), a point developed further in Section 5.2.

The phenological sensitivity to global change is not restricted to *S. alterniflora*, nor to warming alone. A field experiment on the Tibetan alpine meadow demonstrated that warming, precipitation reduction, and nitrogen addition interact to reshape reproductive phenology in a manner dependent on species’ phenological niche and pollination mode: warming advanced flowering onset by 3.5 days and fruiting onset by 3.8 days, but precipitation reduction offset this advancement; warming prolonged flowering and reproduction duration only when interacting with precipitation reduction; nitrogen addition delayed flowering by 2.1 days and fruiting by 1.8 days; and the magnitude of phenological response differed between wind-pollinated and insect-pollinated species (Wang et al., 2025b). These shifts in flowering time and reproductive duration translate directly into altered temporal overlap between invasive plants and their pollinators, herbivores, and seed-dispersing mutualists, which constitutes the temporal dimension of interspecific networks. When an invasive plant’s flowering window desynchronizes from that of native congeners, it reduces pollinator sharing and alters the strength of plant–pollinator links; when its seed-release schedule shifts relative to granivore activity, it modifies plant–seed-predator network topology.

Latitude-wide comparisons reinforce the generality of this mechanism. Along a 20-degree latitudinal gradient of the Chinese coast, seed traits of *S. alterniflora* and the native *Phragmites australis* diverged in 11 of 13 traits with respect to latitude; *S. alterniflora* seed size decreased with increasing latitude, and temperature-dependent climatic variables outweighed soil properties in driving latitudinal trait variation, leading the authors to predict greater fecundity of *S. alterniflora* and an increasing threat to P. australis at higher latitudes under climate warming (Wang et al., 2025a). Experimental confirmation comes from cold-stratification and temperature treatments: longer cold stratification promoted germination rate and index but reduced germination time, and higher germination temperature amplified these effects; the interaction between cold stratification and temperature varied among provenances, with warming magnifying the germination advantage conferred by cold stratification from high- to low-latitude common gardens (Wu et al., 2024). Subtropical and temperate *S. alterniflora* provenances may therefore migrate northward to obtain adequate low-temperature stratification, whereas tropical provenances will lose their germination advantage under ongoing climate warming (Wu et al., 2024). Such changes drive range-wide reconfiguration of suitable establishment zones for the invader, and consequently reshape the native assemblages subject to its invasion.

Collectively, these two directions establish that reproductive and dispersal traits are the mechanistic hinge between global-change drivers and interspecific network reconstruction. Seed functional traits (germination rate, seed mass, pappus loading, terminal velocity) define local dispersal kernels and determine propagule arrival sequence and density, which form the preconditions for chemical and microbial interactions. Extreme climatic factors (climate warming, altered precipitation regimes, nitrogen deposition) modify reproductive phenology, seed allocation patterns and dispersal traits, altering the timing and scale at which invaders colonize native interaction networks (Zhou et al., 2021; Luo et al., 2020). **Figure 3** should therefore be read with this hinge in mind: the arrow from “global change” to “plant-mediated interspecific interactions” is not direct but passes through the quantifiable variables of seed ecology and reproductive phenology. Species distribution models (Section 5.1) that ignore these trait-mediated pathways will systematically overestimate or underestimate invasion spread, a point to which we return in the management synthesis.

## 5 From mechanistic understanding to prediction and management

The ultimate value of an in-depth understanding of the mechanisms behind plant adaptive regulation and interspecific interaction reconstruction under global change lies in enhancing our ability to predict the future state of ecosystems and guiding the development of more predictive, proactive, and resilient ecological management strategies. This requires combining the mechanistic knowledge gained from molecular, individual, to community levels with macroscopic prediction tools and modern technological means, achieving a paradigm shift from “understanding the world” to “intelligently managing the world”. The goal is to develop a novel Intelligent Ecosystem Management framework built upon ecological principles, data-driven approaches and adaptive management to resolve the intertwined complex challenges from diverse global change stressors including biological invasion. (**Figure 4**).

**Figure 4:**
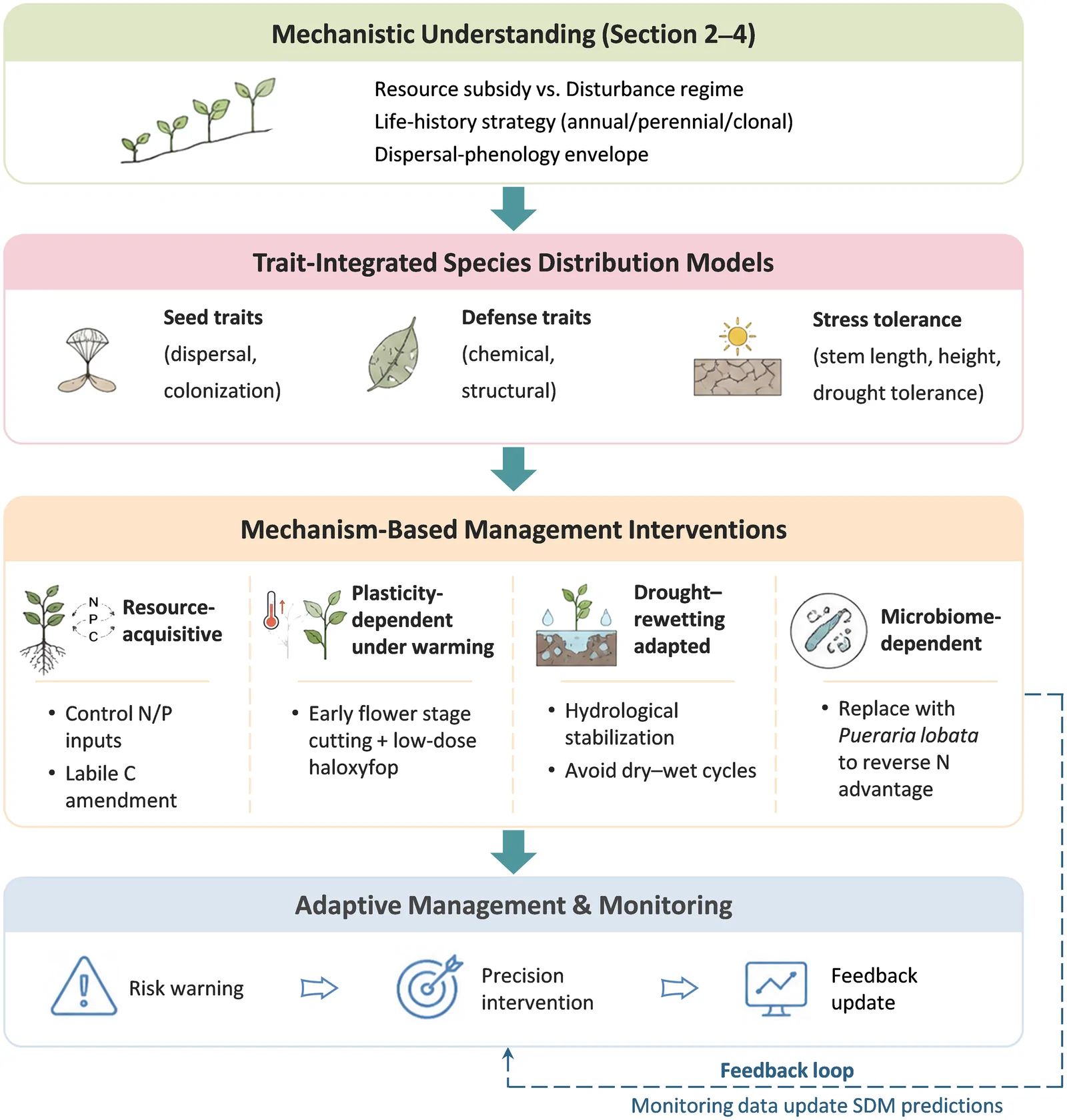
Mechanistic, trait-based framework linking ecological understanding, predictive modeling and adaptive management for invasive plants under global change. This conceptual framework starts with a three-axis mechanistic foundation presented in Sections 2–4, including resource subsidy versus disturbance regime, life-history strategies (annual/perennial/clonal), and the dispersal–phenology envelope. This theoretical basis informs trait-integrated SDMs incorporating seed, defense and stress-tolerance traits (stem length, height and drought tolerance). Model outputs guide four types of mechanism-based management interventions tailored to distinct functional strategies of invaders. After field implementation, control actions enter an adaptive management and monitoring cycle comprising risk warning, precision intervention and feedback update. This module receives field interventions developed from mechanism-based strategies; within this cycle, risk warning guides management prioritization, and post-treatment monitoring enables feedback updates to refine SDM predictions. The dashed feedback loop denotes that post-intervention monitoring data are iteratively used to refine SDM predictions for subsequent risk assessment and control optimization. N represented nitrogen; P represented phosphorus; C represented carbon; SDM represented species distribution model.

### 5.1 Integration of species distribution models and functional traits

Predicting species distribution dynamics under future climate change is an important goal in ecology and conservation biology, with Species Distribution Models (SDMs) being the core tool for this task (Zhang et al., 2023c, 2022). Traditional SDMs primarily predict potential distribution areas based on statistical correlations between species’ current known occurrence points and environmental variables (mainly climatic) (Zhang et al., 2022; Peng et al., 2021). However, such models are essentially descriptive, lacking mechanistic consideration of key biological processes such as species dispersal capacity, adaptive potential, and interspecific interactions, thus having considerable uncertainty in predicting species responses to long-term or severe climate change (Cao et al., 2024; Zhou et al., 2022; Zhang et al., 2022; Peng et al., 2021).

Integrating functional traits into SDMs is a key approach to enhancing model predictive power and mechanistic understanding (Zhou et al., 2021; Zhang et al., 2020a). Functional traits are morphological, physiological, or phenological characteristics that affect individual fitness and respond to environmental changes, such as seed size, germination temperature threshold, drought tolerance, specific leaf area, chemical defense traits, etc (Wang et al., 2020b; Li et al., 2020). Key functional trait dimensions integrated into SDMs include: (1) seed traits (seed mass, dispersal mode), which determine dispersal potential and colonization ability, thereby correcting biases arising from dispersal limitation; (2) defense traits (chemical defenses, structural defenses), which influence resistance to herbivores and pathogens, thereby modulating interspecific competition and distribution patterns (Esser et al., 2025); and (3) stress tolerance traits (specific leaf area, plant height, drought tolerance), which reflect resource acquisition and utilization strategies, directly affecting prediction accuracy under climate change.

In specific modeling practices, MaxEnt remains the most widely utilized approach among prevailing species distribution modeling algorithms, given its robust predictive performance when only species presence records are accessible. By contrast, generalized linear models (GLMs) and generalized additive models (GAMs exhibit optimal performance for datasets containing paired presence-absence observations, while boosted regression trees (BRTs) excel at capturing complex interactions among environmental predictors via ensemble learning frameworks (Sumbh et al., 2025). Model calibration exerts a decisive influence on final predictive reliability. For MaxEnt implementations, taxon-specific tuning through combinatorial tests of feature classes (linear, quadratic, hinge and product) and regularization multipliers is strongly advised to balance model fit and complexity (Esser et al., 2025). In terms of environmental variable preprocessing, predictors with clear mechanistic ecological linkages to focal species should be prioritized, and collinear covariates eliminated where the variance inflation factor (VIF) exceeds a threshold of 10. For example, the systematic variation in ragweed seed germination strategy and chemical composition along temperature gradients directly reflects its adaptation strategies to different climate zones (Zhou et al., 2021; Zhang et al., 2020a). Incorporating such trait-environment relationships into models allows predictions to be based not only on climatic envelope matching but also on the physiological and ecological potential of species to track suitable climates through trait adjustment (phenotypic plasticity) or evolution (local adaptation) (Cao et al., 2024; Zhou et al., 2022).

The core inherent drawbacks of conventional correlative SDMs include the equilibrium assumption (i.e., species fully occupy all environmentally suitable habitats), inadequate consideration of dispersal constraints and interspecific biotic interactions, as well as spatially uneven sampling biases (Sandel et al., 2025). Multiple complementary analytical strategies have been proposed to mitigate these deficiencies. Spatial thinning and environmental covariate filtering algorithms effectively alleviate sampling bias; dispersal-associated predictors (e.g., distances to glacial refugia) can be integrated into modelling pipelines to offset the confounding effects of dispersal limitation; joint species distribution models (JSDMs) and hierarchical models of species communities (HMSCs) further enable quantitative quantification of interspecific association signals. The construction of process-explicit modeling frameworks that simultaneously incorporate dispersal dynamics, demographic turnover and intraspecific adaptive evolution constitutes a pivotal research avenue for elevating the predictive generalizability of distribution projections (Esser et al., 2025; Sandel et al., 2025).

Such “mechanistic SDMs” or “trait-based distribution models” can answer deeper questions, e.g., whether a species responds to climate change through migration or adaptation? What are the limits of its adaptability? Which traits are key to limiting its distribution? (Cao et al., 2024; Li et al., 2022b). Furthermore, integrating interspecific interaction information is also crucial (Liu et al., 2022; Luo et al., 2020). For instance, considering how warming alters herbivorous insect preferences for host plants, or how heavy metal pollution alters plant-natural enemy relationships through elemental defense, can simulate the constraints of biotic interactions on species distributions (Wang et al., 2022b; Liu et al., 2022). Combining functional traits with interaction network data to develop dynamic, multi-species ecosystem models is a frontier direction in ecological prediction for the future (Wang et al., 2024; Xing et al., 2024). This requires interdisciplinary efforts integrating ecology, evolutionary biology, physiology, biogeography, and data science to build more realistic and reliable prediction tools, providing scientific basis for biodiversity conservation, invasive species management, and ecosystem adaptation planning (Song et al., 2023; Yu et al., 2023).

### 5.2 Mechanism-based management: matching interventions to invader typology

Broad management slogans centered on native vegetation restoration and soil remediation overlook the diverse mechanistic invasion pathways elaborated in the preceding sections. The three-dimensional framework established in this study delineates distinct invasion mechanisms: resource-subsidy pressures filter for resource-acquisitive invasive species, altered disturbance regimes favor phenotypically plastic invaders, and divergent life-history strategies shape the structural properties of plant defensive networks. This mechanistic distinction indicates that successful invasion management relies on accurate classification of invader functional types and targeted interventions tailored to their dominant invasion strategies. Accordingly, this study categorizes existing management approaches into four mechanistic modules, each matched with a robust, empirically validated control strategy (**Table 1**).

**Table 1:** Mechanism-based management interventions for invasive plants under global change.

Invader mechanism type	Representative species	Dominant global-change driver	Specific management intervention (empirical basis)	Primary reference
Resource-acquisitive (CO_2_/N-responsive)	*Mikania micrantha*, *Chromolaena odorata*, *Eupatorium adenophorum*	Elevated CO_2_, N & P deposition	Landscape-scale control of N/P inputs; labile C amendment (e.g. sucrose) to immobilise soil N	(Zhang et al., 2024a; Ouyang et al., 2014)
Phenotypic-plasticity-dependent under warming	*Spartina alterniflora*	+1.5 °C growing-season warming	CCutting of invasive plants at early flowering stage + low-dose haloxyfop (150 mg m^-^²)	(Zhang et al., 2025a; Liu et al., 2025)
Drought–rewetting adapted	Annual and perennial invaders under extreme precipitation	Drought–rewetting cycles	Stabilize soil moisture regime; avoid creating periodic dry–wet cycles; restore perennial natives; do not blanket-apply AMF	(Pan et al., 2026)
Microbiome-dependent (rhizosphere N-cycling restructuring)	*Mikania micrantha*	Rhizosphere microbiome & allelochemicals	Replacement control with *Pueraria lobatato* enhance nitrification and reverse N advantage	(Wang et al., 2026; Zeng et al., 2025)

1. Resource-acquisitive invaders, such as *Mikania micrantha* and *Chromolaena odorata*, efficiently convert elevated CO_2_ and soil nitrogen enrichment into superior growth performance. Under elevated CO_2_ conditions, N addition alone increases their relative growth rate by 5.4%, while combined CO_2_–N and CO_2_–P elevations further enhance this growth advantage by 7.5–8.1%, reaching a 14.4% advantage over co-occurring natives (Zhang et al., 2024a). Meanwhile, these invasive plants exhibit a significant reduction in leaf defensive metabolites (17.7–29.0% for lipids and structural carbohydrates) alongside increased accumulation of growth-related compounds, including proteins and non-structural carbohydrates (Zhang et al., 2024a). The management corollary is direct: manipulating resource availability neutralizes the invader’s core advantage. For this mechanism type, the intervention should be deployed under the following operational conditions:

Scenario: Peri-urban wetlands, agricultural margins, and riparian zones where anthropogenic N enrichment (fertilizer runoff, atmospheric N deposition > 20 kg N ha^-1^ yr^-1^) acts as the primary invasion driver.

Action 1, N-source interception: Restrict fertilizer runoff via vegetated buffer strips and intercept N-enriched stormwater through constructed wetlands before it reaches invaded patches.

Action 2, Labile C amendment: Apply labile carbon to promote microbial immobilization of soil N. Recent evidence demonstrates that carbon addition (glucose or sodium acetate at 400–800 g C m^-2^) under simulated tidal conditions significantly decreases soil nitrate nitrogen and reduces the biomass of *Spartina alterniflora* roots, stems and leaves (Chen et al., 2026). This “microbial N competition” approach is particularly effective for resource-acquisitive invaders whose growth is directly coupled to mineral N availability.

Action 3, Sugar application: Reducing N availability by applying sugar eliminated the growth advantage of *Eupatorium adenophorum* over its native congener under both ambient and elevated CO_2_ (Ouyang et al., 2014).

Limitation: Carbon amendment efficacy is contingent on soil microbial responsiveness and may require repeated application; monitoring of soil nitrate dynamics is essential to maintain N below the threshold that sustains invader growth.

2. Plasticity-dependent invaders such as *Spartina alterniflora* pose unique management challenges under climate warming. Experimental warming of 1.5 °C during the growing season boosts shoot biomass and redirects biomass allocation toward reproductive spikes (Liu et al., 2025). This response enhances seed production, seed mass, and seed buoyancy, collectively facilitating both clonal expansion and sexual dispersal (Liu et al., 2025). The key vulnerability exposed by this work is temporal: the invader concentrates reproductive resources in the early flowering stage. The operational protocol for this mechanism type is as follows:

Critical window: Cutting of invasive plants must be timed to the early flowering stage (late June–early July for *S. alterniflora* in mid-latitude salt marshes). Delayed cutting after seed maturation stimulates compensatory tillering and releases viable propagules.

Precision intervention: Cutting of invasive plants at an flowering stage paired with low-dose haloxyfop application (150 mg m^-2^) achieved 98.93% control of *S. alterniflora* by the end of the growing season, reduced seedling emergence in the subsequent year by 93.49–96.48%, and caused no significant shift in soil microbial diversity (Liu et al., 2025).

Enhanced physical protocol: Field experiments in the Yellow Sea coastal wetland demonstrate that cutting combined with black film mulching at the end of the growing season achieves significantly higher control efficacy than cutting alone, while simultaneously facilitating the restoration of native *Suaeda salsa* and maintaining macrobenthos density (Chen et al., 2026). This suggests that cutting + mulching should be the preferred protocol where native vegetation recovery is a co-objective.

Limitation: Haloxyfop application requires careful dosage control to avoid non-target effects on native poaceae; cutting residue must be removed promptly to prevent methane emissions from anaerobic decomposition of dead biomass (Zhang et al., 2025b).

3. Drought–rewetting adapted invaders exploit extreme precipitation regimes. Under drought–rewetting cycles, invasive species produced significantly more above- and below-ground biomass than natives, with annual invaders outperforming perennials in above-ground biomass; rewetting reversed drought suppression and favored invaders, while AMF inoculation reduced growth regardless of invasive status, life cycle, or drought treatment (Pan et al., 2026). The operational protocol for this mechanism type comprises:

Hydrological stabilization: Implement regulated hydrological regimes that maintain stable soil moisture and avoid creating periodic dry–wet cycles in managed landscapes. This eliminates the disturbance signal that invaders exploit.

Native restoration: Pair moisture-regime stabilization with restoration of perennial natives whose deep root storage buffers drought without the rewetting-growth surge seen in invaders.

Contraindication: AMF inoculation is not a universal control lever; its effect sign depends on invader life cycle and drought context, so blanket mycorrhizal augmentation is contraindicated and may inadvertently benefit invaders.

4. Microbiome-dependent invaders (e.g. *M. micrantha*) restructure rhizosphere nitrogen cycling through enzyme activation, microbial recruitment, and allelopathic regulation, creating a “high total N, low mineral N” rhizosphere that favors their own nutrient acquisition (Wang et al., 2026). Targeted management therefore operates on the microbial nexus rather than on the plant directly. The empirically validated intervention is:

Native replacement protocol: Replace the invader with Pueraria lobata to reverse the microbiome-mediated N advantage. After one year of *P. lobata* replacement, soil total nitrogen, ammonium, and nitrate concentrations increased by 109.4%, 78.1%, and 20.3%, respectively, while the net nitrification rate rose by 213.0% (Zeng et al., 2025). The abundance of the AOB-*amoA* gene was also significantly enhanced.

Mechanistic basis: Random forest modeling confirms that comammox clade A.2 and AOB jointly dominate the nitrification process; enhanced ammonia-oxidizing microorganism activity improves soil N conversion efficiency, giving P. lobata a competitive advantage over M. micrantha in nitrogen uptake and accumulation, thereby effectively suppressing M. micrantha regeneration (Zeng et al., 2025).

Operational advantage: This strategy breaks the microbiome-mediated nitrogen competitive lock formed by invaders, avoids non-target side effects associated with conventional broadcast herbicides, and delivers a self-sustaining native-dominated successional trajectory.

In summary, management interventions must be matched to the invader’s dominant mechanism type. A nutrient-cutoff strategy that works against *M. micrantha* will not translate to *S. alterniflora*, whose vulnerability lies in reproductive phenology rather than in resource subsidy. Conversely, flowering-stage cutting, precision-timed for *S. alterniflora*, would be ineffective against a microbiome-dependent invader whose propagation is decoupled from above-ground phenology. The operational specifications provided above include scenario applicability, critical timing windows, auxiliary measures, and known limitations, converting the theoretical framework developed in Sections 2–4 into actionable, site-specific management protocols. Species distribution models (Section 5.1) that incorporate these mechanism-based intervention thresholds will yield more realistic forecasts of management outcomes under alternative global-change scenarios.

## 6 Conclusion and outlook

Global change factors, namely warming, elevated CO_2_, nitrogen deposition, extreme climate events, and heavy metal pollution, do not act independently but converge to reshape plant physiology, species interactions, and ecosystem structure. This review synthesizes evidence across scales to propose that invasion outcomes under global change are governed by three axes: resource subsidy (CO_2_, N), disturbance regime (drought–rewetting, warming amplitude), and life-history strategy (annual vs. perennial vs. clonal). Each axis differentially filters invasive lineages by amplifying or suppressing their characteristic defense network architecture. Annual invaders rely on hormone-autonomous, growth-biased networks that are preferentially amplified by resource subsidies. Perennial invaders couple defense signaling to microbial symbionts, conferring durability under disturbance regimes. Clonal invaders extend defense signals across ramets, enabling spatially distributed stress protection. These architectural differences dictate which global-change driver accelerates invasion and which management intervention will succeed. Seed functional traits and reproductive phenology emerge as the mechanistic hinge connecting global-change drivers to network reconstruction. They determine arrival order, propagule density, and temporal windows of interaction, factors that preconfigure community assembly before chemical or microbial mediation begins. Management must therefore move beyond generic prescriptions. We categorize interventions into four mechanism-specific modules: controlling N inputs for resource-acquisitive invaders, early-flowering-stage cutting for warming-responsive invaders, hydrological stabilization for drought–rewetting adapted invaders, and native replacement that reverses microbiome-mediated nitrogen advantage for microbiome-dependent invaders. Each intervention is tied to an operational scenario, a critical timing window, and known limitations.

Future work should integrate functional trait biogeography, multi-trophic network dynamics, and process-explicit species distribution models into a unified predictive platform. Factorial experiments combining multiple global-change drivers, rather than single-factor designs, are urgently needed to capture the non-additive effects that increasingly define real-world ecosystems. Such an approach will transform mechanistic understanding into actionable, site-specific management under accelerating global change.
